# Wearable Sensor System for Detection of Lactate in Sweat

**DOI:** 10.1038/s41598-018-33565-x

**Published:** 2018-10-26

**Authors:** Luke J. Currano, F. Connor Sage, Matthew Hagedon, Leslie Hamilton, Julia Patrone, Konstantinos Gerasopoulos

**Affiliations:** Johns Hopkins University Applied Physics Laboratory, 11100 Johns Hopkins Rd, Laurel, MD 20723 USA

## Abstract

Increased development of wearable sensors for physiological monitoring has spurred complementary interest in the detection of biochemical indicators of health and performance. We report a wearable sensor system for non-invasive detection of excreted human biomarkers in sweat. The system consists of a thin, flexible, kapton patch (2.5 × 7.5 cm) that can be coated with adhesive and affixed to the skin. The system can be controlled by a cell phone via a near-field communications protocol, charged wirelessly, and the data can be downloaded and displayed in a smart phone app. The system is designed such that the sensing element plugs into a low-profile socket, and can easily be removed and replaced as needed due to saturation or aging effects. As a demonstration case, we examined using an organic electrochemical transistor (OECT) within this system to monitor lactate concentration. Several different methods for optimizing the sensor performance were compared, including altering electrode materials, employing various immobilization techniques, and tailoring operating voltages. Resulting functional response of the lactate oxidase enzyme was compared as a function of the sensor variables. The OECT sensor was shown to have high sensitivity to lactate, however the sensing range is limited to lactate concentrations below approximately 1 mM.

## Introduction

Wearable sensors have emerged as a major area of growth for consumer, sports, and military applications as they offer unique possibilities for *in-situ*, real-time, and non-invasive monitoring of health and performance. Currently, the consumer electronics market is flooded with examples of wearable technology in wrist-worn, textile, or strap-mounted formats, comprising rigid active materials and bulky power sources. Available measurements are generally limited to high-level physiological signals such as temperature, heart rate, skin conductance, and breathing rate. Recent advances in conformal and flexible electronics have resulted in impressive demonstrations of research-grade prototypes with similar physiological measurement capabilities, but radically different form factors^1–6^. At the same time, significant emphasis is being placed on monitoring the presence of analytes in biofluids, thus expanding the type of information that can be gathered from the wearer. Sweat is a prime candidate for interrogation due to its compatibility with non-invasive sampling^7,8^. Several sweat sensing devices have been demonstrated in patch, watch and even temporary tattoo form factors, with the ability to measure analytes such as lactate, glucose, ammonia, ethanol, trace metals, hydration biomarkers, pH and other metal ions present in sweat^7,9–14^. More intricate systems combining measurements of multiple biomarkers and physiological signals on the same platform have also been reported^15–17^, showing the potential for fully integrated autonomous systems.

Despite the exponential progress in the field, several key challenges remain, as discussed in detailed reviews^9,18–20^. Two of these challenges include the sensor’s power source and the operation of the sensing element. The power source problem is critical for the remote, autonomous operation of a wearable system. To date, demonstrations of sensors with on board power^15,21^ have relied on coin cell or other bulky batteries, significantly restricting the final form factor. Lee *et al*. reported a novel approach for embedding chip-scale solid-state Li-ion batteries in flexible systems^22^, but the solution significantly limits the overall available energy of the system. The sensor operation challenge refers to the need for sensitive, selective and stable detection systems. The ideal transduction mechanism is fast, label-free, and allows for significant signal amplification and easy electronic read-out. Depending on the analyte of interest, either immunoassay-based or enzymatic sensing may be preferred, both mechanisms requiring functionalization of the sensor surface with a transduction molecule. When using three electrode cells for electronic readout, a label is often required for adequate signal amplification.

In this work, we present a complete wearable sensor system for monitoring the concentration of lactate in sweat. For on board power, we chose to use a state of the art commercially available thin film lithium-ion battery, integrated directly on a flexible Kapton substrate. Future iterations will incorporate new flexible battery technology^23^. As the transducing element, we chose to use an organic electrochemical transistor (OECT). OECTs are compatible with simple, inexpensive fabrication techniques that are well-suited for flexible electronics. OECTs have extremely high transconductance and can serve as label-free sensors for enzymatic and immunoassay detection methods^24–29^, making them potential universal transduction platform candidates for all types of sweat analytes. In previous demonstrations, OECTs were used as sensing elements connected to external instrumentation. The work herein is the first demonstration of a wearable OECT integrated into a stand-alone system, with on board power and controlling electronics that are specifically designed for low power OECT operation. We use lactate as the analyte of choice to demonstrate proof of concept^30–33^, but the sensor platform can be expanded to multiple analytes. This system could provide significant advantages for health and performance monitoring in low-resource settings.

## Results

### Sensor Design and Working Principle

Our OECT consists of a functionalized gate electrode adjacent to an organic semiconductor source-drain channel (poly(3,4-ethylenedioxythiophen)/poly(4-styrene-sulfonate) (PEDOT:PSS)). The gate electrode and source-drain channel are connected via an electrolyte solution containing mobile ions. Positive voltage applied at the gate electrode drives cations into the PEDOT:PSS channel, effectively de-doping the channel and decreasing the conductivity^34^. In this way, a minute change in the gate potential can be detected as a much larger change in the source-drain current. Furthermore, the degree of de-doping depends on the magnitude of the gate voltage. Therefore OECTs can be applied as sensors for chemical reactions that result in small changes in the gate potential. In this work we exploit the enzymatic reaction between lactate oxidase and lactate.

**Figure 1:**
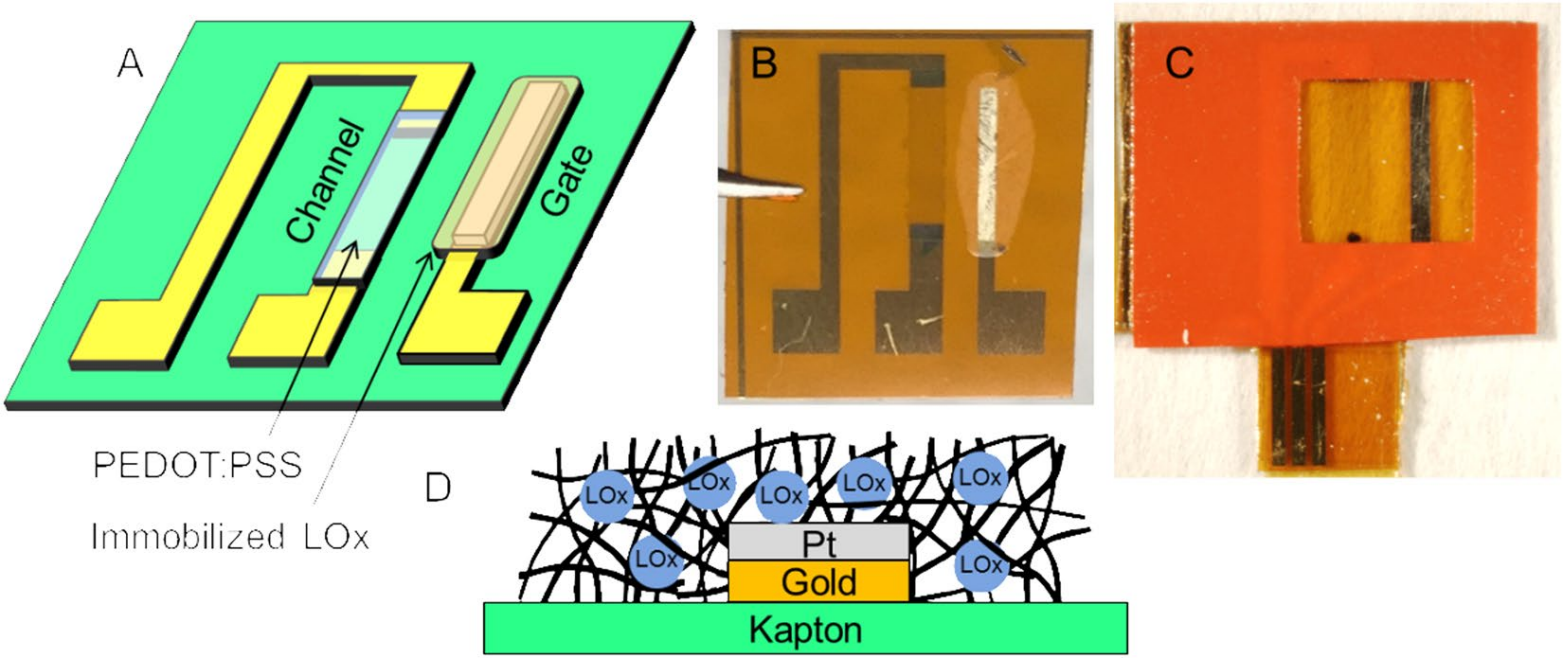
Organic Electrochemical Transistor for sensing lactate in sweat. (**A**) Conceptual drawing of device. (**B**) Photograph of device with platinum gate functionalized with immobilized lactate oxidase (LOx). (**C**) Photograph of device with vinyl sticker applied for confining solution above active gate and channel region of OECT. (**D**) Cartoon of gate cross-section, showing LOx immobilized in notional gluteraldehyde matrix.

Our sensor is pictured in Fig. 1. The pads and interconnects are gold, deposited on a kapton substrate. The gate electrode is either platinum or Prussian blue, coated with lactate oxidase (LOx) immobilized in either a chitosan or gluteraldehyde matrix (see Methods section for immobilization details). The enzymatic reaction of lactate with lactate oxidase produces hydrogen peroxide. The platinum or Prussian blue catalyze the reduction of hydrogen peroxide. The gate electrode donates electrons in this process^35,36^, increasing the gate potential and leading to de-doping of the channel in proportion to the amount of hydrogen peroxide present. The rate of hydrogen peroxide produced is dependent on the rate of the lactate/lactate oxidase reaction, which is in turn dependent on the availability of the lactate oxidase and the concentration of lactate present. Therefore, the sensitivity and range of the sensor is expected to be affected by the amount of lactate oxidase, and the response of the sensor is expected to scale with the amount of lactate present.

**Figure 2:**
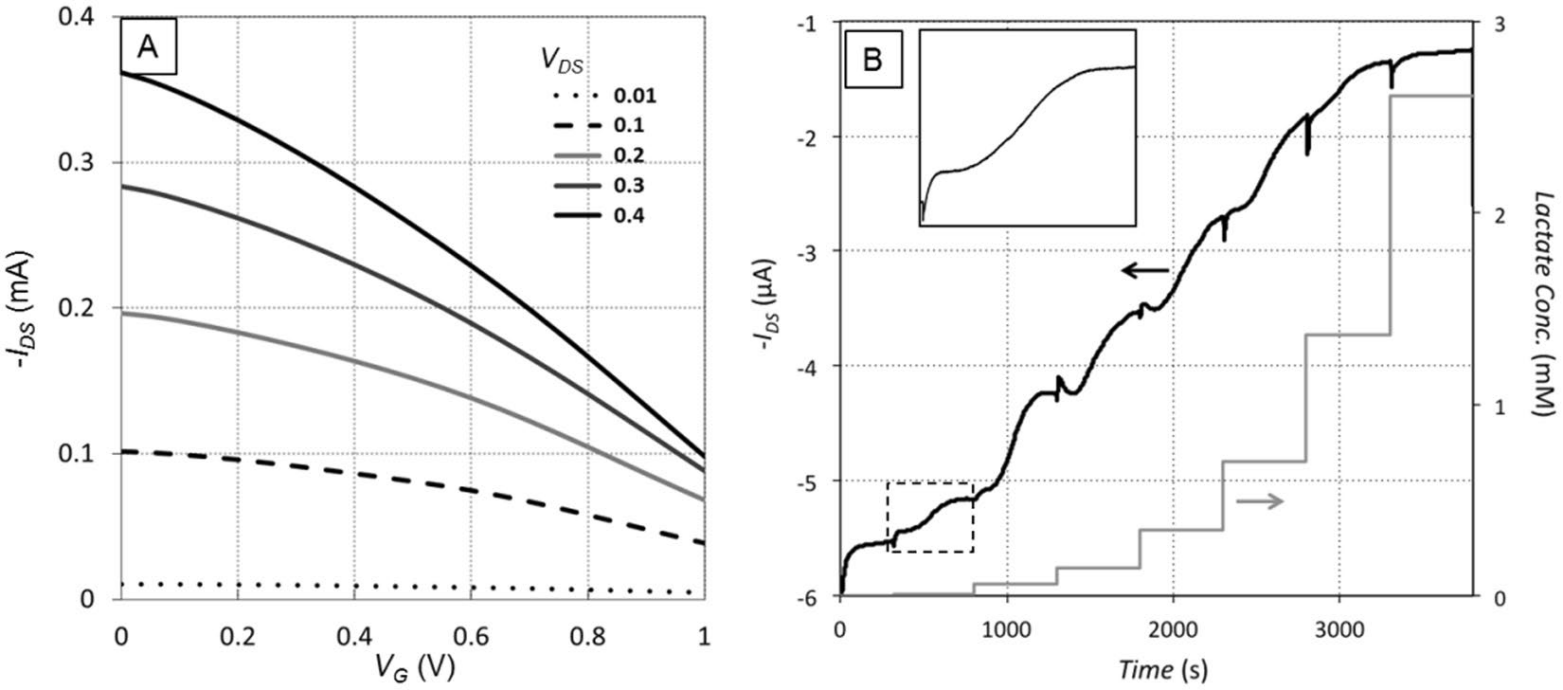
Response of OECT with 40 µL of phosphate buffer electrolyte on top (platinum gate, functionalized with 154 U/mL LOx according to Method A. (**A**) Typical transfer curves from OECT in electrolyte. (**B**) Time response of OECT with multiple additions of lactate. Step increases in concentration represent addition of higher concentration of lactate solution to the droplet on the sensor. Inset in B) is an expanded view of the sensor response to a single addition (dashed box), showing initial steep decrease in current, followed by flattening out, slower secondary decrease, and finally levelling off. The effective concentration in the inset is 11 nM.

The sensors were tested by applying a vinyl sticker that covered the interconnects while keeping the channel and gate electrode exposed (Fig. 1C). A 40 µL droplet of phosphate buffer was applied to the top of the sensor, the drop was held in place over the channel and gate by the hydrophobicity of the vinyl sticker. The channel source was grounded and the drain was biased with a negative voltage *V*_*DS*_. A set of transistor transfer curves was collected by incrementally increasing the gate voltage *V*_*G*_ from 0 to 1 V and recording the drain current *I*_*DS*_ (Fig. 2A). The drain current was recorded as negative because of the negative bias on the drain terminal.

The electrochemical response of the sensor to lactate was evaluated using the same setup as the transfer curves, but with the source-drain bias *V*_*DS*_ and gate voltage *V*_*G*_ fixed, and the drain current was monitored over time. Every 500 seconds a 5 µL drop of lactate solution in phosphate buffer was added to the electrolyte solution covering the sensor. Each successive droplet added contained a higher lactate concentration than the previous drop, typically starting at 1 mM and ending at 100 mM. Taking into account the initial 40 µL of pure buffer, these serial additions result in actual lactate concentration levels of the electrolyte ranging from 0.01 mM to 9.8 mM. A typical time response to multiple serial additions of increasing lactate concentration is shown in Fig. 2B. The characteristic response to an increase in lactate concentration consists of an initial rapid decrease in current magnitude, followed by a flattening out, a second, slower decrease, and finally a levelling off. After the lactate concentration reaches a critical value, the sensor saturates and subsequent increases in lactate concentration do not result in further changes in the drain current. Detectable sensor response was recorded for lactate concentrations as low as 11 nM.

The observed behavior is based on changes in the effective gate potential, dictated by the rate of electron donation by the gate. Although the gate voltage is nominally fixed via a DC power supply, the electron donation results in an effective increase in gate potential. This can be viewed as moving to the right along the transistor transfer curve (Fig. 2A). Since the positive gate voltage causes de-doping of the channel and a reduction in the channel conductivity, moving to higher gate potential decreases the magnitude of the drain current. In our sensor, this means that as the concentration of lactate increases, the drain current decreases via a 3-step process: (1) lactate reacts with lactate oxidase to produce hydrogen peroxide; (2) the gate electrode reduces the hydrogen peroxide, and the effective gate voltage increases; (3) mobile ions in the electrolyte are pushed into the channel and de-dope it, reducing the conductivity. This 3-phase process takes 5–10 minutes from the addition of lactate to reach equilibrium, as the lactate diffuses to constant concentration in the buffer and the chemical reactions reach a stable rate.

The saturation of the sensor at higher concentrations of lactate (Fig. 2B) does not appear to be limited by de-doping in the PEDOT:PSS, as smaller drain currents are often encountered further along in the corresponding transistor transfer curve. Rather, it appears to be limited by reaching a maximum rate in either the enzymatic reaction or catalytic reaction. Further study is needed to determine which reaction is rate limiting and design ways to alleviate this limit and extend the range of lactate concentration that the sensor can discriminate.

### Optimization of sensor performance

**Figure 3:**
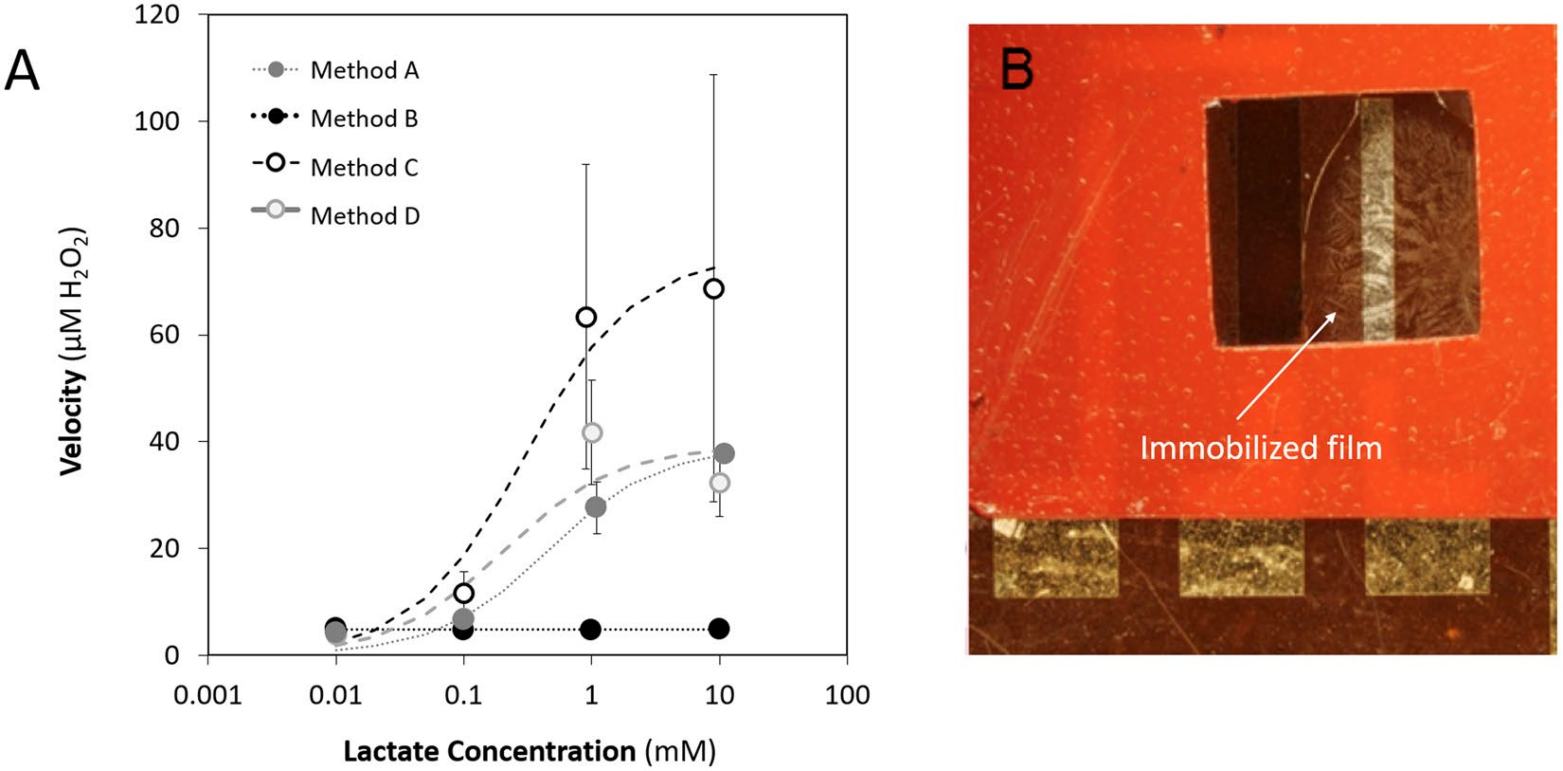
Evaluation of different immobilization methods, BSA and glutaraldehyde (Method A), Chitosan and PVC (Method B), Chitosan alone (Method C) and Chitosan and glutaraldehyde (Method D). (**A**) Effect of Immobilization Method on Enzyme Binding Kinetics. 15.4 U/mL LOx was immobilized to Kapton films using four unique methods. Error bars indicate standard error of the mean. Note that x-axis values have been offset slightly for better visualization of error bars. The error bars for Method D are smaller than the markers. Trend lines represent least squares fit to the Michaelis-Menten equation. Enzyme kinetic data resulting from Method A resulted in the best fit to the model of the methods tested (R2 = 0.9551). (**B**) Stability of immobilization Method A demonstrated with a sample that has been tested, rinsed, and dried multiple times, and stored for more than 6 months in phosphate buffer. The material is still clearly adhered to the substrate.

Immobilization methods from the literature^16,24,37^ were adapted for use with the OECT device. Details on immobilization techniques A, B, C, and D are given in the Methods section. Prior to functionalization, these methods were evaluated for performance under conditions comparable to reported physiological conditions^8^ and that were also presumed to be compliant with the requirements set for optimal electrical sensing. A colorimetric hydrogen peroxide detection assay was utilized to quantitatively measure the oxidation of lactic acid by lactate oxidase. This method for assessing enzyme function is independent of the peroxide reduction reaction and de-doping of the gate, and makes evaluation of the lactate oxidase/lactate interaction at the gate simpler and faster. Each immobilized area was exposed to one concentration of lactic acid at room temperature for 500 s. Raw absorbance data was then collected at 595 nm using a colorimetric peroxide detection assay. Data shown in Fig. 3A have been normalized to a standard curve to determine peroxide concentration and represent two independent experiments, each performed in triplicate, for a total of 6 measurements of each immobilization technique/lactate concentration combination.

Immobilization Method B (chitosan and PVC), was found to produce little to no H_2_O_2_ in the colorimetric assay as can be seen in Fig. 3. In drop-casting the lactic acid over this immobilized area it was apparent that the surface was extremely hydrophobic, which clearly limited penetration of lactate into the film, therefore limiting the enzymatic reaction. This result was repeatedly observed across the tested range. Method C was procedurally similar to Method B except that it did not include the addition of PVC. The results from this method, while demonstrating enzyme function, resulted in highly variable peroxide levels across experiments. This is evident in the large error bars for Method C in Fig. 3, indicating that Method C is not a useable immobilization method. The mechanical properties of Method D immobilization layers (chitosan hydrogel paired with a glutaraldehyde cross-linker) were inconsistent. In some instances, all of the immobilized enzyme delaminated during the washing step before use. This resulted in no observable H_2_O_2_ production from these samples. For this method, only valid trials (defined as those in which the immobilization layer remained intact throughout testing) are included in Fig. 3. Among these valid trials, reproducible levels of hydrogen peroxide were measureable, however the lack of mechanical stability indicates Method D is not a useable immobilization method. Based on these observations, it was concluded that the composition of the chitosan-based immobilization matrices, as implemented, led to either limited stability and/or efficiency of the lactate oxidase enzyme.

The final immobilization technique was comprised of a hydrogel containing bovine serum albumin (BSA) rather than chitosan. Similar to Method D, Method A also used glutaraldehyde as a cross-linker. The results obtained from this formulation were more consistent in comparison to those of the other formulations tested, and there were no issues with delamination of the immobilized LOx, even after multiple rinsing and testing cycles (Fig. 3B). The data from all samples were analyzed by non-linear regression and least squares fit to the Michalis-Menten model of enzyme kinetics. Samples created using Method A provided the best fit to the model (R^2^ = 0.95501), and greatest *V*_*max*_ with least data variability. While the other methods did not fit the Michalis-Menten model due to issues noted above, the least square curves are still included as an aid to the eye. The *V*_*max*_ is theoretically attained when an enzyme is saturated by an infinite concentration of substrate, given the affinity of the enzyme-substrate interaction^38,39^. The goodness of fit to the model, high *V*_*max*_, and low data variability indicate the functionality and stability of the bound enzyme using this immobilization method. From a practical standpoint, Method A also provided desirable qualitative mechanical properties – resulting in a matrix that remained intact and attached to the substrate even after numerous washes. For these reasons, this technique for LOx immobilization was chosen for further exploration in an attempt to optimize enzyme performance on the sensor.

**Figure 4:**
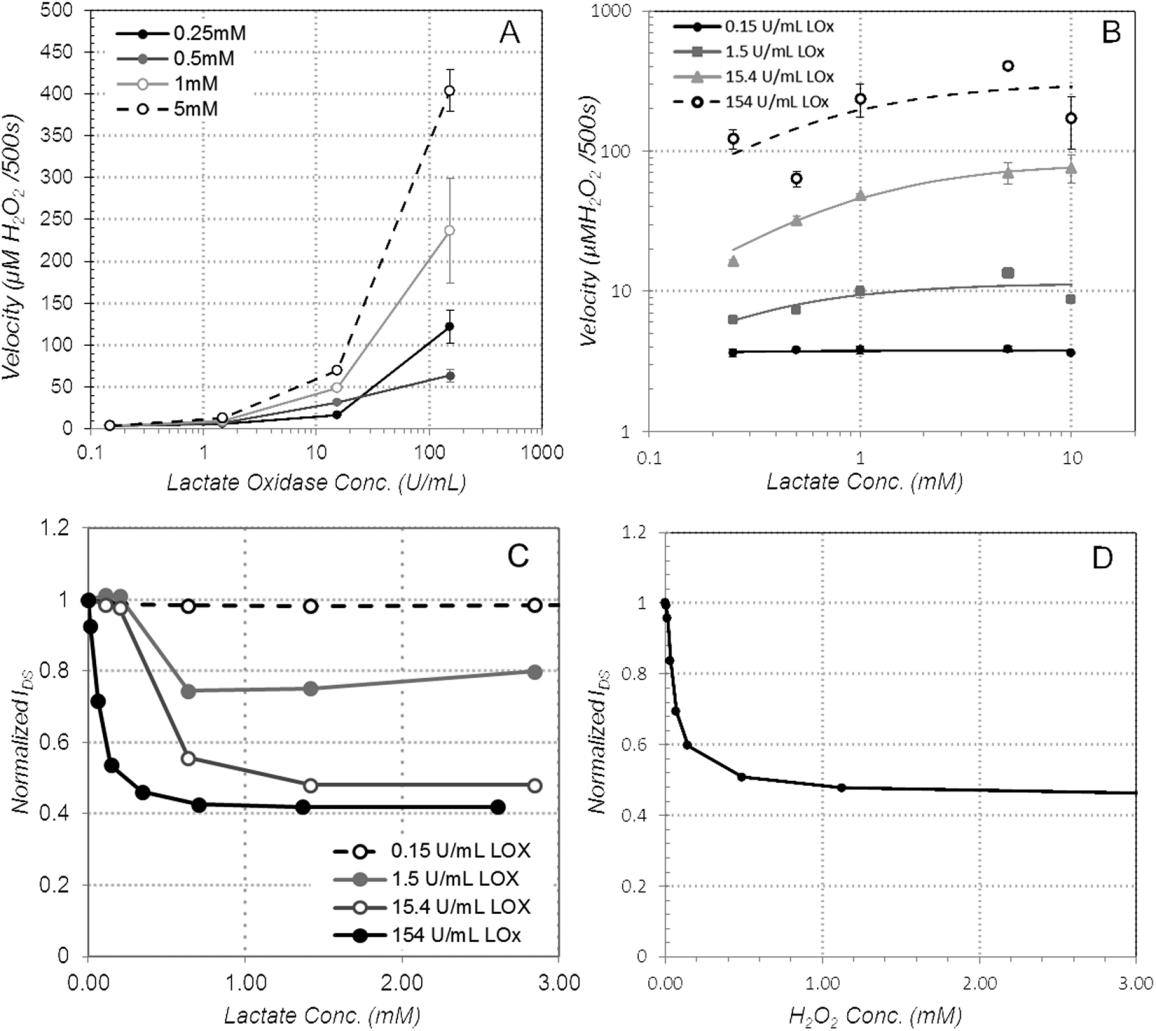
The effect of varying LOx and lactate concentration on sensor response, (**A**) H_2_O_2_ production as a function of LOX concentration, (**B**) H_2_O_2_ production as a function of lactate concentration, (**C**) drain current as a function of LOx and lactate concentration (platinum gate, *V*_*G*_ = 0.4, *V*_*DS*_ = −0.4 V) and (**D**) drain current as a function of H_2_O_2_ concentration (platinum gate, 154 U/mL LOx concentration, *V*_*G*_ = 0.4, *V*_*DS*_ = −0.4 V).

We next studied the role of the lactate oxidase concentration on sensor response. We prepared samples with varying concentration of lactate oxidase in the immobilization matrix over three orders of magnitude (0.15–154 units of LOx per mL), using immobilization Method A. Absorbance at 595 nm was measured using the colorimetric hydrogen peroxide assay to quantify enzyme velocity. Increasing the concentration of LOx in the Method A formulation led to an increase in H_2_O_2_ production at a given concentration of lactate, as expected (Fig. 4A). Similarly, increasing the amount of lactate present also increases the H_2_O_2_ production for a given concentration of LOx (Fig. 4B). The data from all samples were analyzed by non-linear regression and least squares fit to the Michalis-Menten model of enzyme kinetics, resulting in estimated *V*_*max*_ values of 3.8, 11.4, 82.7, and 305.4 µM H_2_O_2_/500 s for the 0.15, 1.5,15.4, and 154 U/mL LOx concentration conditions (with corresponding R^2^ of 0.029,0.549, 0.886, and 0.388).

These results were confirmed on a set of OECT devices functionalized with 5 µL of Method A formulation, using the same 0.15–154 U/mL LOx concentrations (Fig. 4C). The devices were tested by putting a 40 µL droplet of phosphate buffer on the OECT (exposing just the gate and channel – interconnects were covered with a vinyl sticker). Successive additions of phosphate buffer/lactate solution were added in the same way as Fig. 2B, and the change in current recorded with each addition (Fig. 4C). At the lowest LOx concentration (0.15 U/mL), the introduction of lactate has no measureable effect on the drain current. At higher LOx concentrations, the drain current decreases as lactate is added, up to a saturation point. Beyond this point, further lactate additions do not produce further changes in the drain current. For the conditions tested, the magnitude of current modulation increases with oxidase concentration, to nearly 60% for the 154 U/mL case, agreeing with the colorimetric assay result that showed that peroxide generation increases with oxidase concentration. The maximum concentration of lactate that can be discriminated also appears to increase with oxidase concentration, although the increase appears slight - even at the highest LOx concentrations the lactate sensing range is effectively limited to <1 mM. As observed in the colorimetric assay, with more lactate oxidase available, there are more reaction sites for lactate, so the maximum achievable rate of hydrogen peroxide production should also increase. At first glance, this trend does not appear to fully translate to the OECT response, however.

To separate the OECT electrical response from the enzymatic reaction kinetics, an OECT with 154 U/mL LOx immobilized on the gate was tested directly by serial additions of hydrogen peroxide at increasing concentrations (Fig. 4D). This experiment bypasses the enzymatic reaction, isolating the sensor response to illuminate the maximum amount of hydrogen peroxide that can be reduced at the gate, and the corresponding de-doping levels reached. After each addition of H_2_O_2_, the sensor was allowed to stabilize for 500 s before adding more peroxide, matching the incubation time for the colorimetric assay. The results show that the OECT response saturates at peroxide concentrations above about 0.5 mM. As measured in the colorimetric assay, *V*_*max*_ for the lower LOx concentrations (1.5 and 15.4 U/mL) was found to be 11.4 and 82.7 µM of H_2_O_2_ production per 500 s, respectively. These are both well within the linear range of OECT drain current modulation, so the response of the sensor to the peroxide produced is not expected to be a limiting factor. For the highest concentration of LOx, the enzymatic *V*_*max*_ was found to be 305 µM of H_2_O_2_ production per 500 s. This is close enough to the H_2_O_2_ saturation point in Fig. 4D that reduction of peroxide at the gate could be a limiting factor in the OECT response to lactate in this case. However, since the OECT showed similar saturation levels for all of the three highest LOx concentrations, it appears that some other factor may be limiting the lactate sensing range.

**Figure 5:**
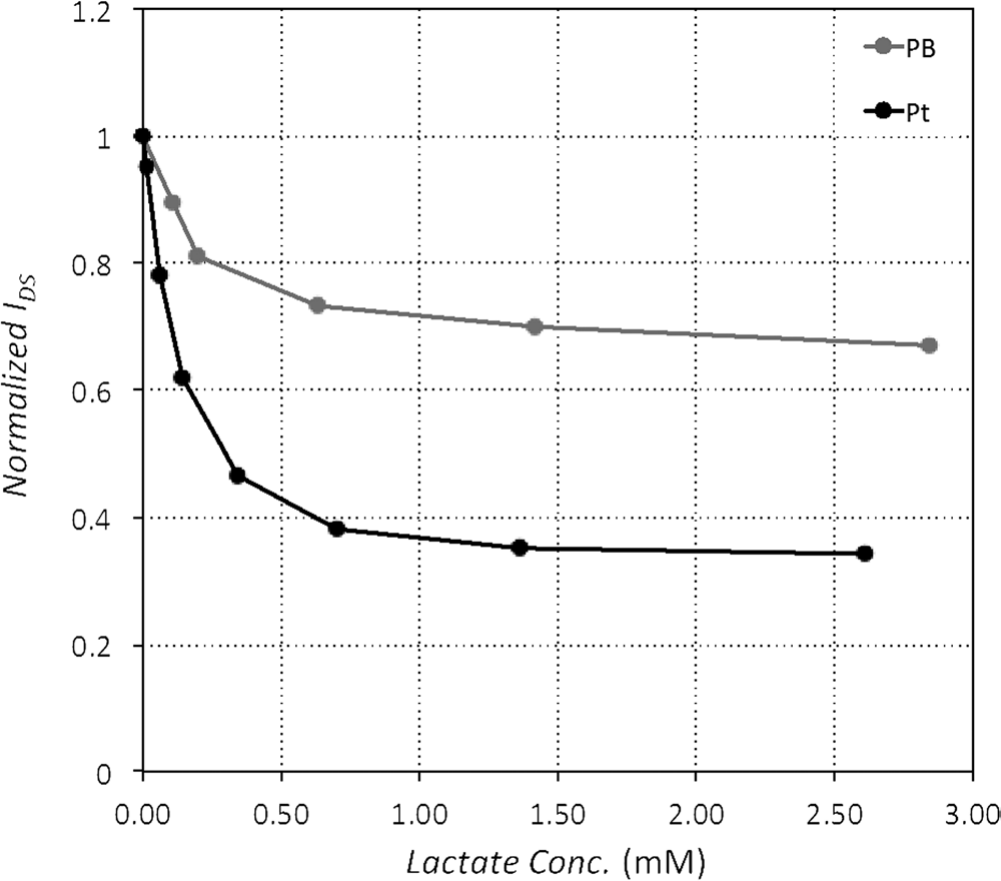
Comparison of platinum and Prussian blue gate electrodes. Each device was functionalized with 154 U/mL LOx immobilized on the gate using immobilization method A.

We next evaluated OECT performance with different gate electrodes, platinum and Prussian blue, to determine the better gate material. Immobilization method A was used to immobilize 154 U/mL LOx over the gate of each device. The results are shown in Fig. 5. Both platinum and Prussian blue catalyze the reduction of hydrogen peroxide, resulting in the donation of electrons from the gate. Platinum can also reduce oxygen, resulting in the possibility of donating more electrons per hydrogen peroxide molecule. The observed change in drain current was consistently higher (approximately 2x higher) for platinum gate electrodes than Prussian blue under the same operating conditions. However, the maximum lactate concentration sensed before saturation was similar for both. We conclude that platinum gate yields better sensitivity with no loss in sensing range, so platinum was used for all subsequent tests.

**Figure 6:**
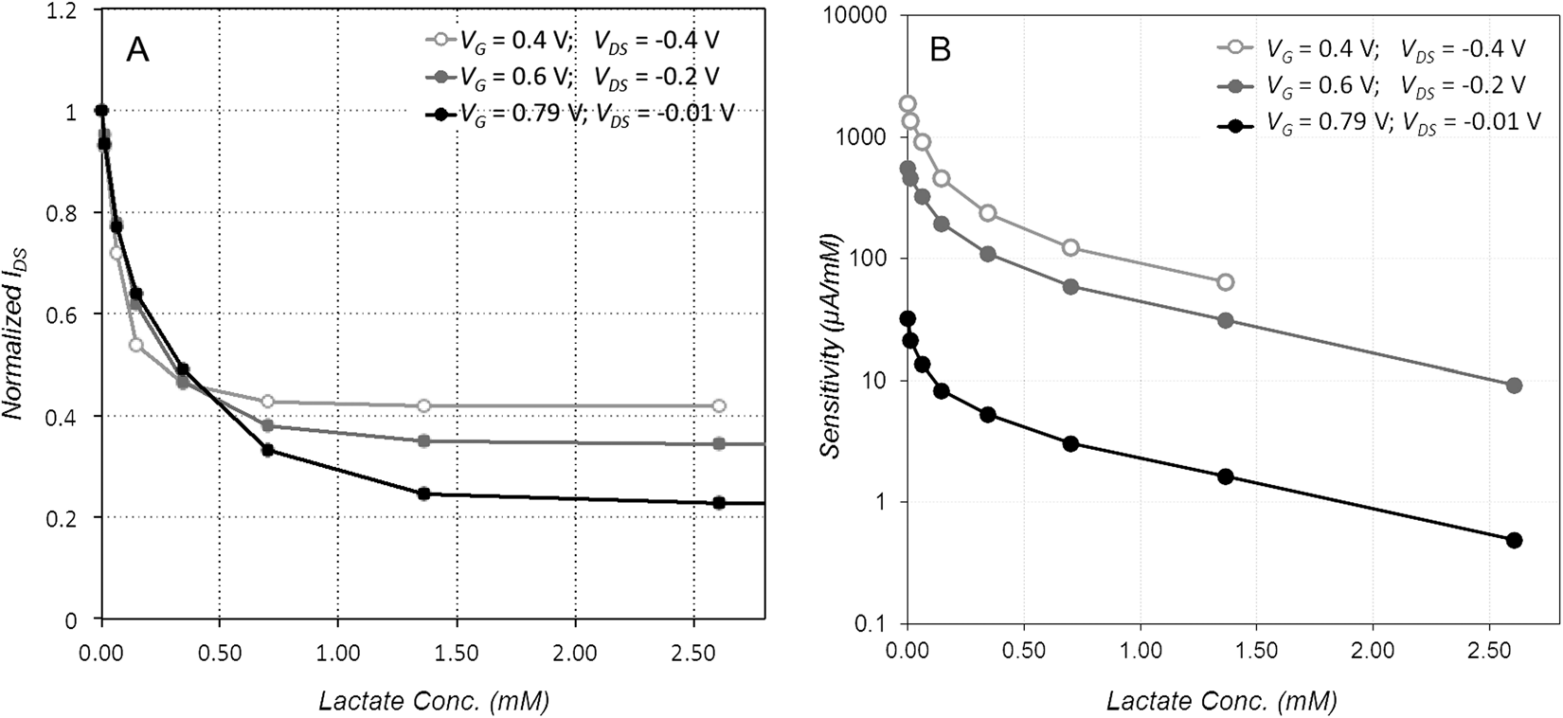
Effect of gate voltage on OECT sensor performance. (**A**) Normalized drain current as a function of lactate concentration for different bias conditions. (**B**) Sensitivity as a function of lactate concentration for different bias conditions. Each curve was produced with a fresh platinum gate device, functionalized with 154 U/mL LOx immobilized on gate using Method A.

We next studied the impact of the gate and drain potential on sensor performance. Three different OECTs were made with platinum gates and 154 U/mL LOx was immobilized on each using immobilization method A. The transfer curve (Fig. 2A) shows that increasing gate voltage results in progressively steeper drain current changes, which is expected to lead to higher sensitivity to small changes in the gate voltage. The maximum potential drop in the OECT is between the gate (which is at a positive bias) and the drain (negative bias). In order to offer a fair comparison, this maximum potential drop was kept fixed at 0.8 V. Three different gate voltage conditions were evaluated, with the drain voltage modified to keep the gate/drain potential fixed (Fig. 6). Under these conditions, larger *Vg* increases the amount of modulation for a given lactate concentration, and extends the linear range slightly (Fig. 6A). Because we are decreasing *V*_*DS*_ while increasing *V*_*G*_, the drain current is also reduced, so total power consumption decreases as well. Therefore, we conclude that higher gate voltage and lower (absolute value) drain voltage improve sensor performance. However, these conditions also decrease the raw sensitivity of the sensor in µA/mM (Fig. 6B), so this must be balanced with noise and precision of the current measurement circuit.

### System Design

**Figure 7:**
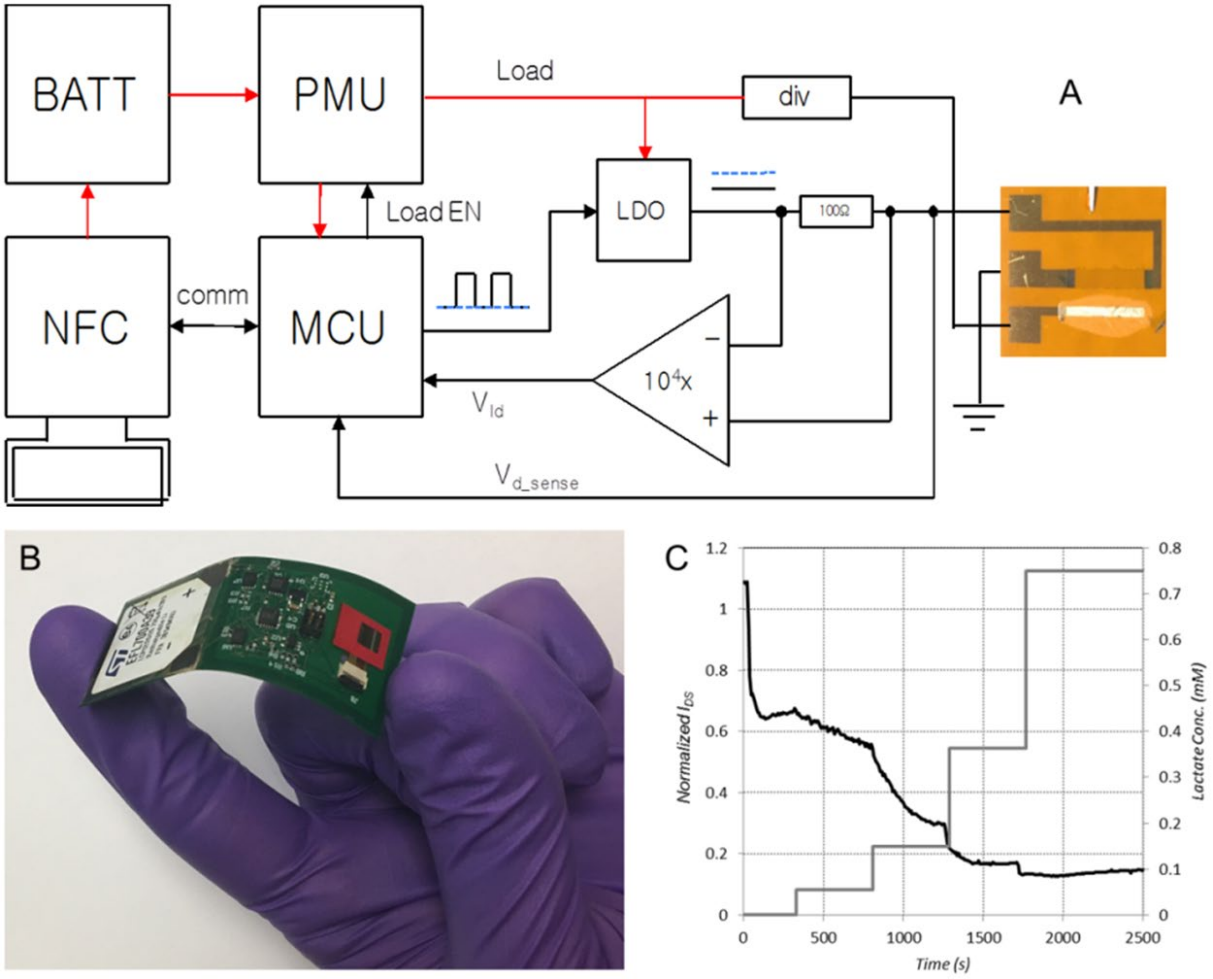
Wireless patch (**A**) system block diagram, (**B**) flex board implementation, and (**C**) response to lactate additions as collected by wireless patch electronics (LOx was dissolved in phosphate buffer solution rather than immobilized on gate for this experiment).

To demonstrate the application of the lactate sensor, a flexible sensing platform capable of collecting sensor data as well as exfiltrating the data to a smartphone app was developed. The block diagram and a picture of the flex board implementation is shown in Fig. 7. The platform consists of a microcontroller (MCU) (ATtiny24A, Atmel), an NFC interface (AS3955, AMS), a battery (EFL700A39, ST Microelectronics), a power management IC (PMU) (TPS82740A, Texas Instruments), and the sensing circuitry (Fig. 7A). The MCU controls the state of the system (sleep, sensing, or communications mode) based on commands received from the NFC interface. The MCU also handles transfer of data from the onboard non-volatile memory through the NFC interface. The NFC interface handles the protocol for communication with an app running on an Android smart phone and concurrently harvests power from the communication signal to charge the on-board battery through its internal voltage regulator.

Sensing of the OECT requires measurement of *I*_*DS*_ under the conditions of a bi-polar voltage between the drain and the gate. For this reason, the sense circuit contains an inverting voltage regulator to supply a negative voltage which is then regulated down by the MCU to supply the desired voltage at the drain. A current sense amplifier is placed in line from the amplifier output to the OECT drain and the output of the amplifier is read and recorded to non-volatile memory by the MCU.

While in sleep mode, the patch consumes 24 µW, and while in sensing mode it consumes 2–4 mW (varies depending on the sensor state). Data recorded from a sensor test with serial additions of increasing lactate concentration are shown in Fig. 7C. Due to the instability of the settling time of the sensor after application of power, the sensing circuit must remain active during operation rather than sleeping between measurements. Because of this, operational lifetime is limited to about 40 minutes using the 700 µAh ST Microelectronics commercial battery.

## Discussion

We have demonstrated a fully-integrated wearable sensor system for detection of analytes in sweat. Our system consists of a 2.5 × 7.5 cm electronics patch which can collect, store and transmit data from the sensor. In principle, the sensor can be designed to sense many different types of biomarkers. As a proof of concept, we demonstrated this with an OECT functionalized with lactate oxidase to detect lactate; this approach could be multiplexed to include other sensors in the future. While the sensitivity of the OECT was very good, we did find that there are some limitations to the sensing range (generally saturates at lactate levels above 1 mM) and the time response to changes in concentration is on the timescale of several minutes, so other sensor types warrant investigation for abundant analytes. However, the OECT remains an interesting choice for biomarkers that are present in sweat only at trace concentrations, or that are otherwise difficult to detect.

The electronic patch developed in this work can be coated with a medical-grade adhesive and worn much like a smart adhesive bandage. If a reusable adhesive is used, it can be repeatedly stuck and unstuck. One advantage of our architecture is that the sensing element can be easily removed and replaced if it saturates or ages in a way that affects performance, while the electronics can be reused indefinitely.

For wearable lactate sensors in the literature, the sensitivity is typically orders of magnitude lower than our sensor. Jia *et al*., reported linear sensor response for lactate concentrations from 1–20 mM, with sensitivity of 0.64 µA/mM for a 3-electrode cell configuration^12^. Khodagholy *et al*., report sensor response from 10–100 mM lactate concentration, for an OECT configuration using a ferrocene mediator. They do not report sensitivity, but from their data it also appears to be on the order of 0.6 µA/mM^40^. Gao *et al*., report a linear response from 5 mM to 30 mM, with sensitivity of 0.22 µA/mM, for a 3-electrode configuration^15^. In contrast, though our sensor response is nonlinear, sensitivity can be as high as 1.9 mA/mM, depending on bias conditions (Fig. 6B).

Many works in the literature reporting continuous sensors for lactate or similar metabolites, report data on sensor response to serial concentration additions without clearly defining how the concentration is determined. This can lead to difficulty in comparing the response of two different reported sensors. Specifically, if one begins an experiment exposing the sensor to lactate at a specific concentration, then adds additional lactate, there are multiple ways in which to report the resulting concentration. Reporting the concentration of the addition seems to be common, but this approach is misleading. Once the additional lactate solution is mixed with the initial volume, the actual resulting concentration lies somewhere between the two, depending on the relative volumes of each. We believe the most appropriate way to report the concentration is to calculate the resulting concentration based on the volume and concentration of each addition – which is the approach adopted in this work. In fact, even this is not exact; additional confounding factors exist due to evaporation of water and consumption of lactate in the enzymatic reaction. We measured the evaporation rate of the phosphate buffer droplet at the starting volume (40 µL) as well as the typical maximum volume after additions (80 µL). The average evaporation rate over the course of our experiments is 0.7% per minute. Based on our peroxide assay experiments (Fig. 4A,B) consumption of lactate is approximately 10–50 µM per minute (0.1–0.4 mM/500 s) for our preferred LOx concentration of 154 U/mL. Over the course of an experiment, evaporation works to increase the actual concentration, while consumption of lactate works to decrease the actual concentration. We do not account for these confounding factors in our graphs since they are difficult to quantify precisely. However, the counteracting trends predict overall small to moderate changes in lactate concentrations for our typical test procedure (<10% for lactate concentrations <1 mM; increasing to 22% for lactate concentrations of 2.8 mM). It is worth noting that at elevated temperatures (e.g. to mimic body temperature and maximize enzyme function), evaporation becomes dominant and changes to the experimental approach are suggested (see Supplemental Fig. S1).

A related issue that merits some discussion is defining the concentration of lactate oxidase reported. Rather than reporting the concentration of the stock LOx solution, we believe it is more representative to report the concentration of the full immobilization mixture deposited on the device. Therefore, the LOx concentration throughout this study has been reduced by the volume of gluteraldehyde, chitosan, BSA, and other reagents accordingly. This convention makes it easier to ensure the total amount of LOx deposited on the device is the same, regardless of immobilization technique.

## Methods

### Materials

Ethylene glycol (≥99%), 4-Dodecylbenzensulfonic acid (≥95%), Acetic acid (99.7%), bovine serum albumin (BSA), chitosan, glutaraldehyde (25% wt), hydrogen peroxide (H_2_O_2_, 30% wt in H_2_O), L-(+)-lactic acid (≥98%), phosphate buffer solution (PB, 0.1 M), poly (vinyl chloride) (low molecular weight) (PVC), and tetrahydrofuran (anhydrous, 99.9%) were purchased from Sigma-Aldrich (St. Louis, MO). (3-Glycidoxypropyl)trimethoxysilane (97%) was purchased from Alfa Aesar (Tewksbury, MA). Lactate oxidase (106 U mg^−1^) was purchased from Toyobo Corp. (Osaka, Japan). Phosphate buffered saline (PBS, 1X sterile) was purchased from VWR International (Philadelphia, PA). 500FPC Kapton film was purchased from American Durafilm (Holliston, MA). Clevios PH 1000 conductive polymer was purchased from Heraeus Precious Metals North America (Conshohocken, PA). Carbon Graphite Ink containing Prussian Blue (P/N C2070424P2) was purchased from Gwent Group (Pontypool, UK).

PEDOT:PSS stock solution was mixed immediately before use, combining 40 mL Clevios PH 1000 conductive polymer with 10 mL ethylene glycol, 8 drops of 4-Dodecylbenzensulfonic acid, and 500 µL of (3-Glycidoxypropyl)trimethoxysilane. The mixture was then sonicated for ten minutes and deposited on substrates the same day.

### Enzyme Immobilization Methods

Prior to functionalization of the OECT devices, parameters of enzyme immobilization were evaluated for optimal performance in dynamic range. Three immobilization techniques adapted from the literature^16,24,37^ were employed, in addition to one technique that was designed to combine characteristics from two of these sources^24,37^. Stock solutions of all necessary reagents were made as follows: 1000 U/mL LOx in PBS, 1 M acetic acid, 27.5 mg/mL PVC in THF, 25 mg/mL BSA in PBS, and 2.5% wt glutaraldehyde in deionized water (di-H_2_O). Each immobilization method was carried out on a 1.5 cm^2^ kapton film placed into the well of a 6-well plate.

Method A^37^: The stock LOx solution was diluted to 100 U/mL before being added to the BSA solution in a 1:5 v/v ratio. Glutaraldehyde (2.5% wt) was then added to the LOx-BSA mixture in a 1:12 v/v ratio. The solution was mixed by gently vortexing before 5 µL of the homogenous suspension was drop-cast onto a square of kapton film and allowed to dry at room temperature overnight. The immobilization layer was washed with PBS to remove any excess enzyme. The kapton film were then stored in a sealed 6-well plate at 4 °C until use.

Method B^16^: Chitosan (5 mg/mL) was added to 1 M acetic acid and then stirred for one hour, or until the chitosan was completely dissolved. During this time, stock LOx solution was diluted to 31 U/mL and then enough lyophilized BSA was added for a concentration of 10 mg/mL. This mixture was added in a 1:1 v/v ratio to the chitosan solution. Devices were incubated overnight as described in Method A. The following morning a suspension of PVC in THF was prepared (27.5 mg/mL). 3 µL was drop-cast over the immobilized area. After at least three hours of drying at room temperature, each immobilization layer was washed and stored as described in Method A.

Method C^24^: A 50 mM acetic acid solution was prepared from the stock. Chitosan (5 mg/mL) was added and then this solution was left to stir for one hour, or until the chitosan was completely dissolved. During this time, stock LOx solution was diluted to 31 U/mL. The chitosan and LOx solutions were then combined in a 1:1 v/v ratio. The solution was mixed and drop cast onto kapton squares and devices were incubated and stored as described in Method A.

Method D: The immobilization solution was prepared exactly as in Method C, followed by the addition of glutaraldehyde (2.5% wt) in a 1:100 v/v ratio. The solution was mixed and drop cast onto kapton squares and devices were incubated and stored as described in Method A.

For the experiments comparing each immobilization method, each of the solutions prepared by the above methods contained the same final concentration of LOx delivered onto the substrate −15.4 U/mL. For experiments comparing LOx concentrations, Method A was used with varying concentrations of LOx stock solution – 1000 U/mL, 100 U/mL, 10 U/mL, and 1 U/mL, with the stock solution diluted appropriately in phosphate buffer. These correspond to final concentrations actually delivered to the device of 154 U/mL, 15.4 U/mL, 1.5 U/mL, and 0.15 U/mL.

### Characterization of Immobilization Techniques and Enzyme Performance

To test the dynamic range of each immobilization method (results in Fig. 3), lactate was prepared in PBS at varied concentrations: 0.01 mM, 0.1 mM, 1 mM, and 10 mM. For every test, enough lactate solution (70 µL) was added to the surface of the film to completely cover the immobilization surface. A Pierce Quantitative Peroxide Assay Kit (Thermo Scientific #23280) was used to quantify the amount of hydrogen peroxide (H_2_O_2_) being produced by LOx in each of the immobilization preparations. A standard curve was prepared in a 96-well plate according to the instructions of the kit for each quantitative assay that was performed. After 500 seconds, the entire sample volume was drawn off the film and stored in a 1.5 mL sample vial at room temperature for no more than ten minutes. 20 µL of this sample volume was added to 200 µL of working reagent. Each sample was prepared in triplicate. Blanks were prepared by adding 20 µL of di-H_2_O, lactate at the highest tested concentration, or working reagent to 200 µL working reagent. The 96-well plate was sealed, placed on a plate shaker, and incubated at 25 °C for at least one hour, but not exceeding twelve hours. Absorbance at 595 nm was measured using a plate reader (Tecan Infinite M1000 PRO and Tecan Safire II).

Method A was chosen to measure the effect of varying the amount of LOx on lactate turnover (Fig. 4). The Method A formulation was not altered, except for the concentration of LOx solution used in the recipe. Four LOx concentrations were evaluated: 1, 10, 100, and 1000 U/mL, which correspond to 0.15, 1.5, 15.4 and 154 U/mL overall concentration of LOx in the final mixture. Lactate was prepared at five different concentrations: 0.25 mM, 0.5 mM, 1 mM, 5 mM, and 10 mM. Each combination was prepared in triplicate and tested using the peroxide assay kit in the same way as above.

### Sensor Fabrication

Sensors were fabricated on 500FPC 0.005″ thick kapton film. The kapton was first pre-treated with a 170 °C bake for 1 hour to ensure no shrinkage would occur during later processing. The film was then exposed to O_2_ plasma for 30 s at 100 W RF and pressure of 100mT to improve adhesion. Chrome/Gold (10 nm/100 nm thickness) interconnects and pads were then patterned on the film via photolithography, ebeam evaporation and liftoff using Futurrex NR9-3000PY photoresist. For platinum gate devices, the Cr/Pt (10 nm/100 nm thickness) gate was patterned and deposited on top of the gold trace using photolithography, ebeam evaporation, and liftoff with the same NR9-3000PY resist. The channel shape was patterned using S1827 photoresist spun at 4000 rpm for a 2.7 µm thick coating. PEDOT:PSS stock solution was applied using a dropper on top of the resist layer and the film was spun at 900 rpm for 55 s, then baked on a hotplate at 110 C for 10 min. The spin/bake step was repeated to increase the thickness and conductivity of the gate. Liftoff of photoresist and PEDOT:PSS film outside of the channel area was completed by soaking in ethanol for ~2 min, then adding ultrasonic agitation for ~30 s. Following liftoff, the PEDOT:PSS layer was annealed at 140 C for 60 min. For devices with Prussian Blue gates, the Gwent Group carbon graphite ink was screenprinted on top of the gold trace in the gate area.

## Electronic supplementary material

Supplementary Infortmation
