# Supplementary material for: Wearable Sensor System for Detection of Lactate in Sweat
Source: Sci Rep. 2018 Oct 26;8:15890. doi: 10.1038/s41598-018-33565-x (PMC6203741; doi:10.1038/s41598-018-33565-x)
Supplement: Supplementary file 1 — Supplementary Infortmation [file 41598_2018_33565_MOESM1_ESM.pdf]

Supplementary Information

Wearable Sensor System for Detection of Lactate in Sweat

Luke J. Currano[1], F. Connor Sage[1], Matthew Hagedon[1], Leslie Hamilton[1], Julia Patrone[1], and Konstantinos Gerasopoulos[1*]

[1] Johns Hopkins University Applied Physics Laboratory, 11100 Johns Hopkins Rd, Laurel, MD 20723, U.S.A.

*Corresponding author, email Konstantinos.Gerasopoulos@jhuapl.edu

Temperature comparison

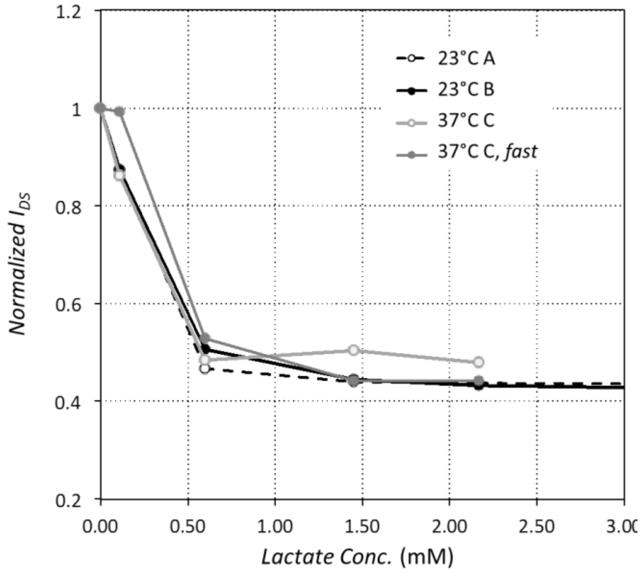

Figure S1. Effect of temperature on OECT response to lactate. Each curve represents a separate OECT sensor with platinum gate and 1000 U/mL LOx immobilized on the gate using immobilization method A. The repeat of the 37C trial used shorter times in between the first two additions to minimize electrolyte evaporation.

Because this device is intended for a wearable sensor, we compared performance at room temperature (23° C) and at core body temperature (37° C). The maximum skin temperature is ordinarily lower than the core body temperature and higher than room temperature, so these two conditions bracket the expected range of operation of the sensor. Four separate OECT's were made with platinum gate electrodes and functionalized with 154 U/mL LOx immobilized on the gates using immobilization method A. The results are shown in Figure S1. The only observed change in the response at elevated temperature was a visibly higher evaporation rate of the electrolyte, which effectively increases the lactate concentration slowly over time even when no new lactate is being introduced. This appears in Fig. S1 as a slightly lower apparent saturation point of the sensor. This trial was therefore repeated with the first two additions at shorter time intervals to limit evaporation over the course of the experiment. These first additions have not fully stabilized before the next addition is made, so the recorded drain current is not the steady-state value. Therefore the most accurate response at 37° C is a composite of the first experiment and the second experiment, using the early portion of the first and the late portion of the second. This composite matches very well with the 23° C trials, showing that the device can be used in contact with the skin without changing performance.
